# Hybrid Micro-/Nanoprotein Platform Provides Endocrine-like and Extracellular Matrix-like Cell Delivery of Growth Factors

**DOI:** 10.1021/acsami.4c01210

**Published:** 2024-06-18

**Authors:** Hèctor López-Laguna, Penelope M. Tsimbouri, Vineetha Jayawarna, Ioanna Rigou, Naroa Serna, Eric Voltà-Durán, Ugutz Unzueta, Manuel Salmeron-Sanchez, Esther Vázquez, Matthew J. Dalby, Antonio Villaverde

**Affiliations:** † Institut de Biotecnologia i de Biomedicina (IBB), Universitat Autònoma de Barcelona, Barcelona 08193, Spain; ‡ Departament de Genètica i de Microbiologia, Universitat Autònoma de Barcelona, Barcelona 08193, Spain; § Centro de Investigación Biomédica en Red de Bioingeniería, Biomateriales y Nanomedicina, Instituto de Salud Carlos III, Barcelona 08193, Spain; ∥ Centre for the Cellular Microenvironment, School of Molecular Biosciences, College of Medical, Veterinary and Life Sciences, Mazumdar-Shaw Advanced Research Centre, University of Glasgow, Glasgow G11 6EW, U.K.; ⊥ Centre for the Cellular Microenvironment, Division of Biomedical Engineering, James Watt School of Engineering, Mazumdar-Shaw Advanced Research Centre, University of Glasgow, Glasgow G11 6EW, U.K.; # Institut de Recerca Sant Pau (IR SANT PAU), Barcelona 08041, Spain

**Keywords:** secretory granules, microparticles, drug delivery, growth factors, smart topographies

## Abstract

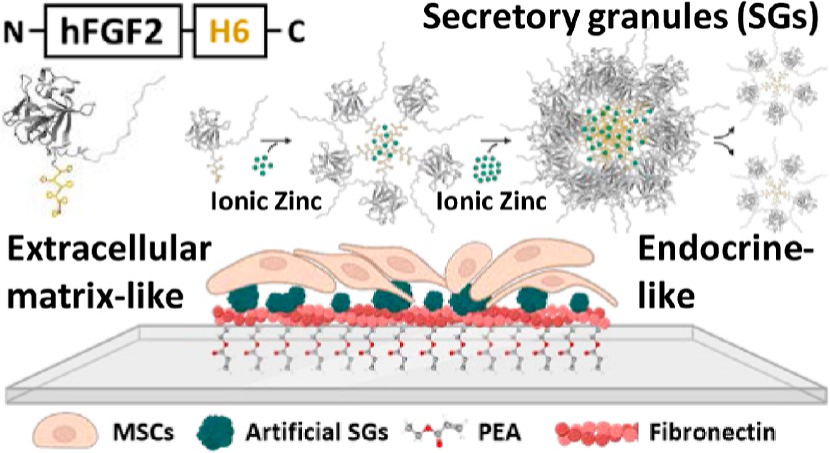

Protein materials are versatile tools in diverse biomedical fields. Among them, artificial secretory granules (SGs), mimicking those from the endocrine system, act as mechanically stable reservoirs for the sustained release of proteins as oligomeric functional nanoparticles. Only validated in oncology, the physicochemical properties of SGs, along with their combined drug-releasing and scaffolding abilities, make them suitable as smart topographies in regenerative medicine for the prolonged delivery of growth factors (GFs). Thus, considering the need for novel, safe, and cost-effective materials to present GFs, in this study, we aimed to biofabricate a protein platform combining both endocrine-like and extracellular matrix fibronectin-derived (ECM-FN) systems. This approach is based on the sustained delivery of a nanostructured histidine-tagged version of human fibroblast growth factor 2. The GF is presented onto polymeric surfaces, interacting with FN to spontaneously generate nanonetworks that absorb and present the GF in the solid state, to modulate mesenchymal stromal cell (MSC) behavior. The results show that SGs-based topographies trigger high rates of MSCs proliferation while preventing differentiation. While this could be useful in cell therapy manufacture demanding large numbers of unspecialized MSCs, it fully validates the hybrid platform as a convenient setup for the design of biologically active hybrid surfaces and in tissue engineering for the controlled manipulation of mammalian cell growth.

## Introduction

Because of their mechanical stability, proteins take, among many other roles, scaffolding functions that in the extracellular matrix (ECM) support the structure and assist the positioning of cells, tissues, and organs, as well as signaling roles found in growth factors (GFs). Indeed, the ECM can act as a scaffold for GFs, presenting them on solid phases and driving more efficient cell stimulation at lower concentrations.^1^ In this context, a significant fraction of artificially engineered protein materials, including particles, layers, fibers, and complex matrices, seek to mimic the complex functionality of the natural ECM.^2−8^ Assisted by protein engineering, protein materials offer enormous functional and structural versatility that allows the incorporation of novel activities of interest (for instance, catalysis or precise cross-molecular binding), based upon precise design approaches.^9,10^ The ability to tune and adapt these properties allows envisaging their development toward clinical applications provided the fabrication process can be made regulatory compliant.

Among the spectrum of clinically appealing protein materials, secretory granules (SGs) from the mammalian endocrine system release peptide hormones, offering control of the cellular milieu.^11^ As with many other structures in nature, they are nontoxic functional amyloids^12−15^ that act as both protein reservoirs and protein-releasing structures. In these depots, peptide chains cluster together through the coordination of cationic Zn and solvent-exposed histidine residues or histidine-rich segments.^16,17^ Taking the inspiration from the protein-clustering properties of divalent cations^18^ and by exploiting the versatility in the engineering of histidine-rich segments in recombinant proteins,^19^ we have developed an approach to fabricate, in vitro, microscale protein depots with time-sustained protein-releasing properties. This is done from pure protein and by using a simple protein–metal coordination protocol.^20,21^ The resulting materials are similar in microscale size and structural composition to natural SGs^22^ and also to bacterial inclusion bodies,^23−25^ protein aggregates naturally occurring in recombinant bacteria when actively producing foreign polypeptides. Although the protein-releasing activities of inclusion bodies make them appealing as time-sustained drug-delivery systems,^26^ their heterogeneous composition and recalcitrant contamination with bacterial cell components prevent them from entering into clinical studies. Interestingly, the nontoxic amyloidal protein occurring in the synthetic SGs^25^ confers them sufficient mechanical stability to be conveniently handled and applied as regular microscale materials.

Being still emerging biomaterials, the secretion properties of artificial SGs have already been robustly validated in oncology. The subcutaneous administration of a particular SG composition has resulted in the release of cytotoxic protein nanoparticles targeted to cancer cells and in the selective destruction of tumor tissues.^27^ This is because clustering as SG does not impair the biological activity of the building block proteins that remain functional even in the case of complex enzymes.^28^ However, apart from the secretion of bioactive proteins, the mechanical stability of SGs should also provide scaffolding properties. In the present study, we have explored the performance of SGs releasing a His(x6)-tagged version of the hFGF2 (hFGF2-H6) as functional topographies on the growth and differentiation of human mesenchymal stromal (or stem) cells (MSCs). hFGF2-H6 was initially selected for the present proof of concept because its biological activity had been preserved, both when the factor is produced in recombinant form as bacterial inclusion bodies^29^ or when it is aggregated in vitro by the addition of ionic Zn.^30^ The experimental setting up was approached by further draw on the natural design of the ECM, where structural proteins such as fibronectin (FN) have cryptic binding sites that open when the protein is under tension in fibrillar conformation.^31^

To mimic this, we have developed a simple polymer coating, namely, poly(ethyl acrylate) (PEA), where, upon absorption, FN molecules elongate and form nanonetworks, revealing FNIII_12–14_ known to bind GFs, including hFGF2. FN also contains the cell adhesion RGD domain at FNIII_9–10_.^32−35^ The solid-phase GF binding to open FN and GF presentation in synergy to integrin binding sites is considered to potentiate GF potency.^33^ Thus, aiming to expand the functionality and clinical potential of SGs as an emerging category of protein materials, we have utilized a cell microenvironment comprising PEA-organized FN with synthetic hFGF2-H6-releasing SGs for a better presentation and further enhancement of the cell response to the sustained release of the GF.

## Results

**Figure 1:**
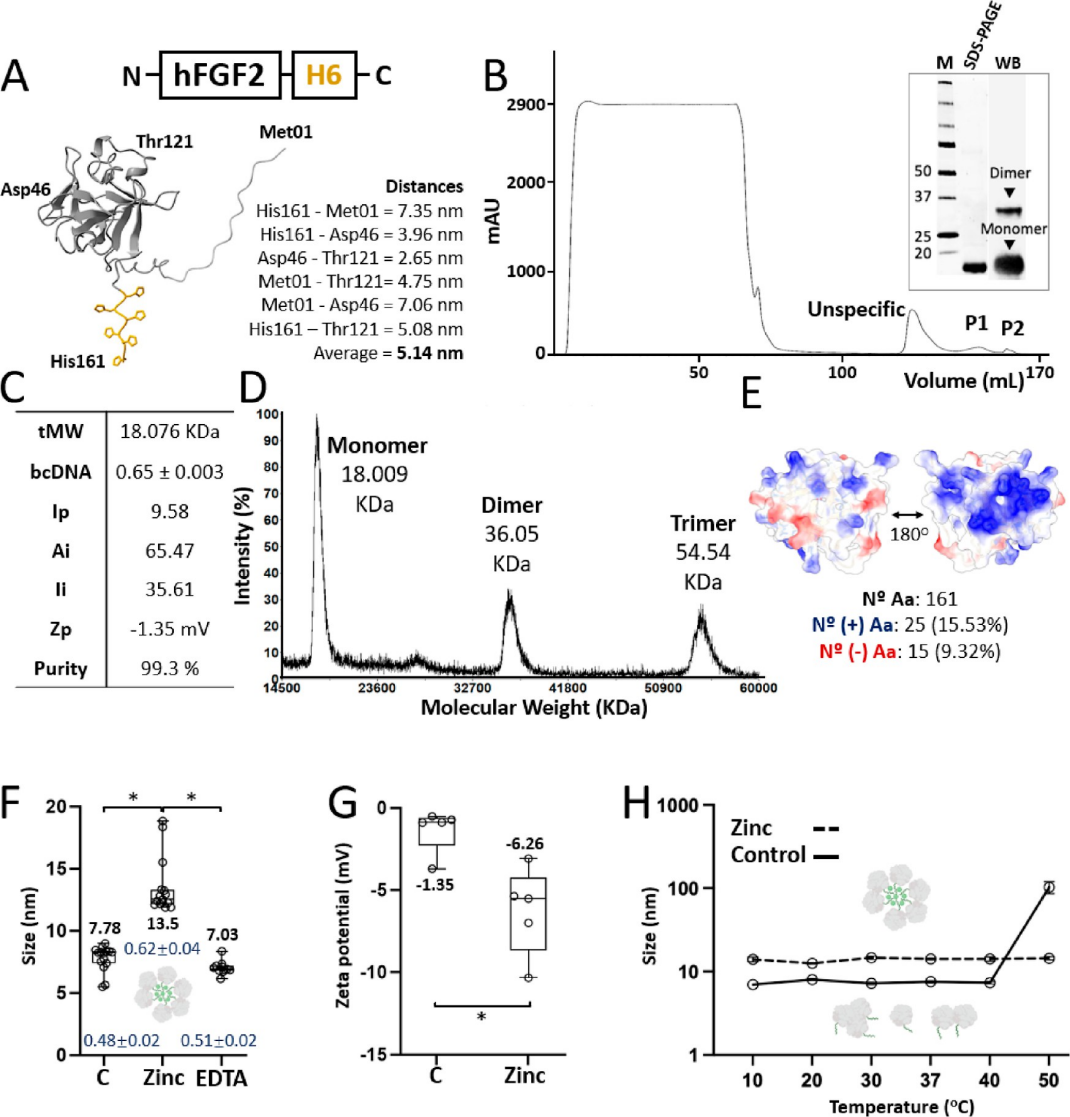
Physicochemical characterization of purified hFGF2-H6. (A) Modular representation of recombinant hFGF2-H6 protein from the N- to C-terminus. Bottom. 3D structure prediction by the Alpha fold. The hexahistidine tag H6 is displayed in yellow. Distances in nm between edging amino acid residues are also shown (namely, histidine 161, threonine 121, methionine 1, and aspartic acid 46). An average monomeric size was afterward calculated in silico. (B) Protein purification chromatogram expressed as mAU (milli absorbance units) vs volume in mL. The protein was eluted in two (P1 and P2) populations. Protein integrity and purity levels are displayed in the inset by SDS-PAGE and WB. (C) Protein physicochemical properties showing theoretical molecular weight (tMW), measured DNA content (bcDNA), isoelectric point (Ip), aliphatic index (Ai), instability index (Ii), measured zeta-potential (Zp), and calculated purity levels. (D) MALDI-TOF spectra are represented by the intensity (in %) vs the molecular weight (in kDa). Monomeric, dimeric, and trimeric structures were detected. Peak numbers refer to the respective MW. (E) Surface charge distribution was predicted using the 3D structure from panel A, displaying both protein sides. Positive amino acid residues are displayed in blue and negatively charged in red. The corresponding percentages are also indicated. (F) Volume size distribution (VSD) in nm of soluble hFGF2-H6 in the presence of 0.4 mM zinc II (Zn^2+^) and after the subsequent addition of 1 mM of EDTA. Polydispersion index values (PDI; dark blue) are additionally displayed for each condition, providing protein size dispersion within the sample and their respective errors. To provide additional size intensity data supporting the VSD already presented, the respective intensity values for each sample are as follows: C (7.89 ± 0.24 nm), zinc (15.25 ± 0.58 nm), and EDTA (7.01 ± 0.18 nm). (G) Zeta-potential in mV of soluble hFGF2-H6 in the presence or absence of 0.4 mM Zn^2+^. (H) VSD in nm of soluble hFGF2-H6 upon increasing temperature (from 10 to 50 °C) in the presence or absence of 0.4 mM of Zn^2+^. C refers to the control protein free of additives for panels (F–H). Data are expressed as mean ± standard error (SE). Statistical significance (*p* < 0.05) is represented as (*).

A hexahistidine (H6) tail was genetically fused to the C-terminus of hFGF2 to confer cation-mediated clustering properties to the protein with a minimal impact on the hFGF2 structure and function. This was done to allow the in vitro fabrication of self-disintegrating protein granules out of the recombinant protein. The H6-tagged hFGF2 version, namely, hFGF2-H6, is a regularly folded polypeptide with molecular dimensions up to 7 nm (Figure 1A). This protein was produced in *Escherichia coli* and purified through immobilized metal affinity chromatography, resulting in protein isolates in which a monomeric form was especially abundant, sided by minor amounts of a dimeric version (Figure 1B). The molecular mass of hFGF2-H6 was as theoretically predicted (18 kDa), and the production and purification steps rendered a purity level estimated as 99.3%. Also, the protein preparations were stable and free of DNA contaminants (Figure 1C). The MALDI-TOF analysis revealed a minor occurrence of a trimeric form (Figure 1D), which was not observed by Western blot (WB) upon denaturing SDS-PAGE (Figure 1B). Such an intrinsic tendency to oligomerization might be favored by the polar distribution of electrostatic charges on the protein surface (Figure 1E) and deemed as positive regarding the controlled protein clustering using divalent cation-histidine coordination. In this context, cationic Zn added to the protein solution (peaking at the monomer size of around 7 nm) at equimolar amounts with histidine residues from H6, generated homooligomeric nanoparticles with a hydrodynamic size of 13.5 nm (Figure 1F). This is similar in range to other assembling protein constructs obtained by the same procedure.^36^ In fact, the oligomeric nanoparticles are intermediates in the cation-mediated clustering process of His-tagged proteins that end up in the formation of microscale SGs.^37^ These nanoscale materials were further disassembled by EDTA to the original size (7 nm; Figure 1F), proving the reversibility of the assembly process and the critical intervention of the cationic Zn in it. The nanoparticles showed a more negative *Z*-potential than the plain soluble hFGF2-H6 (Figure 1G), which indicates enhanced solubility in contrast to the more aggregation-prone plain polypeptides. The assembled protein was also more thermally resistant than the unassembled version, which aggregated between 40 and 50 °C (Figure 1H). All of these data confirmed that the nanoscale oligomers were structurally more stable than their building blocks.

**Figure 2:**
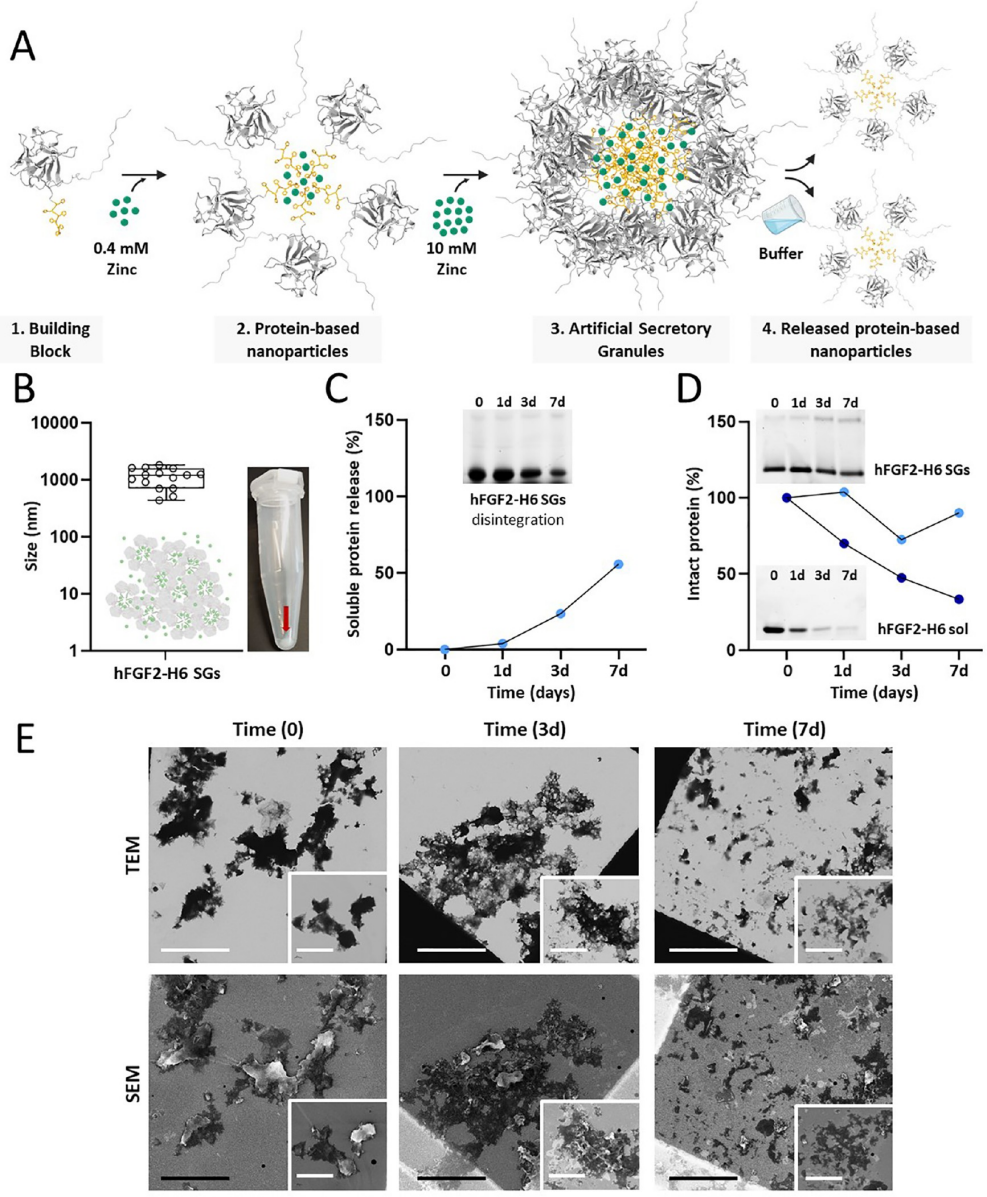

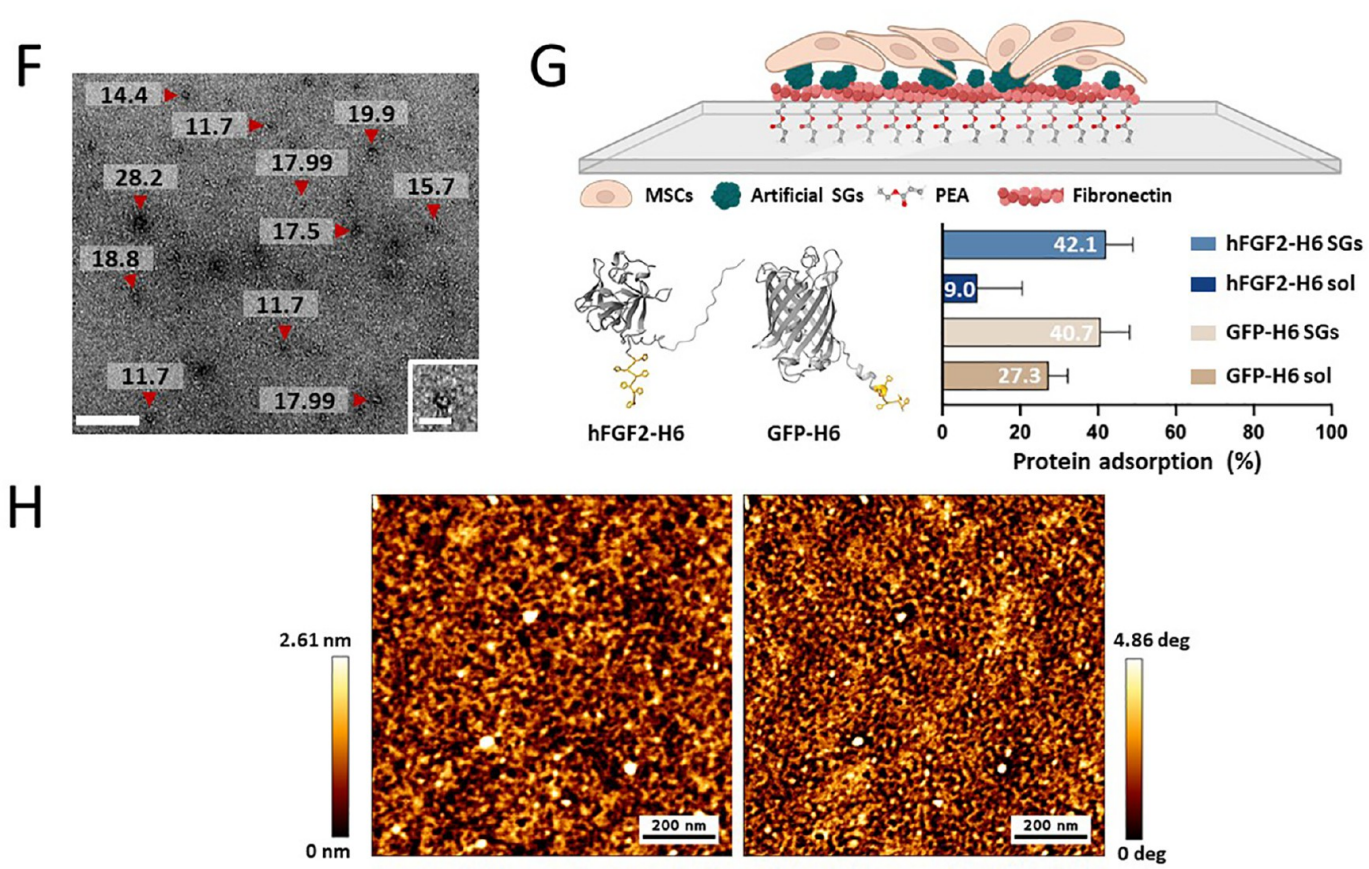
Formulation and physicochemical characterization of hFGF2-H6 SGs. (A) Schematic representation of the manufacturing process from building blocks (step 1) to SGs (step 3). This is done for the further release of soluble nanoparticles (step 4). The histidine tag is displayed in yellow and zinc in green. (B) VSD of hFGF2-H6 SGs as determined in different DLS lectures. Right: Picture displaying the resultant SGs pellet. (C) Cumulative fraction (in percentage) of soluble hFGF2-H6 released from SGs upon incubation at 37 °C for 7 days. Inset. SDS-PAGE shows the disintegration of SGs at 37 °C through the remaining insoluble protein. (D) Percentage of remaining intact protein after SGs incubation at 37 °C for 7 days. Insets: SDS-PAGE shows protein degradation of SGs (top) and soluble (bottom) hFGF2-H6 at 37 °C. (E) Imaging of disintegrating hFGF2-H6 SGs in storage solution (166 mM NaCO_3_H) by simultaneous transmission electron microscopy and scanning electron microscopy (TEM, up; and SEM, down) upon 37 °C incubation at different time points (0, 3, and 7 days). Long scale bars (both black and white) refer to 10 μm, and inset scale bars refer to 5 μm. (F) Imaging of released hFGF2-H6 nanoparticles from SGs by transmission electron microscopy (TEM). The scale bar refers to 100 nm. Nanoparticle size in nm is displayed on top. In the inset, a closer view of the nanoparticle architecture is shown. The scale bar in the inset refers to 25 nm. (G) Schematic representation of the functionalization of PEA–FN surfaces with artificial SGs. MSCs are to be added on top. At down-left, 3D structure prediction by the Alpha fold of hFGF2-H6 and GFP-H6 proteins. GFP-H6, constructed under the same modular pattern than hFGF2-H6, was used as a control nonfunctional protein. The histidine tag is displayed in yellow. At down-right, protein adsorption in the percentage of both H6-tagged hFGF2 (blue) and GFP (brown) SGs (pale colors) and soluble protein (sol; dark colors) on top of PEA–FN surfaces. White numbers represent the protein adsorption in percentage for each condition. (H) Atomic force microscopy (AFM) high (left) and phase (right) images of the FN nanonetwork on PEA-coated glass coverslips.

As stated above, nanoparticles assembled through Zn-His coordination are expected to be intermediates in a clustering process that conduces to higher-order micrometer particles (Figure 2A). The ability of these microscale materials to release the intermediate nanoparticles renders them appealing as secretory protein depots, and these principles were tested for the H6-tagged hFGF2 version. At molar excess amounts of ionic Zn (10 mM), hFGF2-H6 clustered as discrete particulate materials of around 1 μm in size that pelleted as insoluble material under low-speed centrifugation (Figure 2B). Following resuspension in a physiological buffer, a gradual leakage of the full-length protein into the soluble fraction was determined from the proteolytically stable (Figure 2D) microparticles over at least 7 days (Figure 2C), with the granules observed as dynamic disintegrating structures (Figure 2C inset and 2E) and still maintaining greater levels of intact protein in comparison to soluble hFGF2 (Figure 2D). TEM analysis of the soluble fraction confirmed the occurrence of nanoparticles (Figure 2F), with dimensions ranging from 11 to 20 nm, similar to those of the oligomeric intermediate materials, which participated in the construction of the granules (Figure 1F).

**Figure 3:**
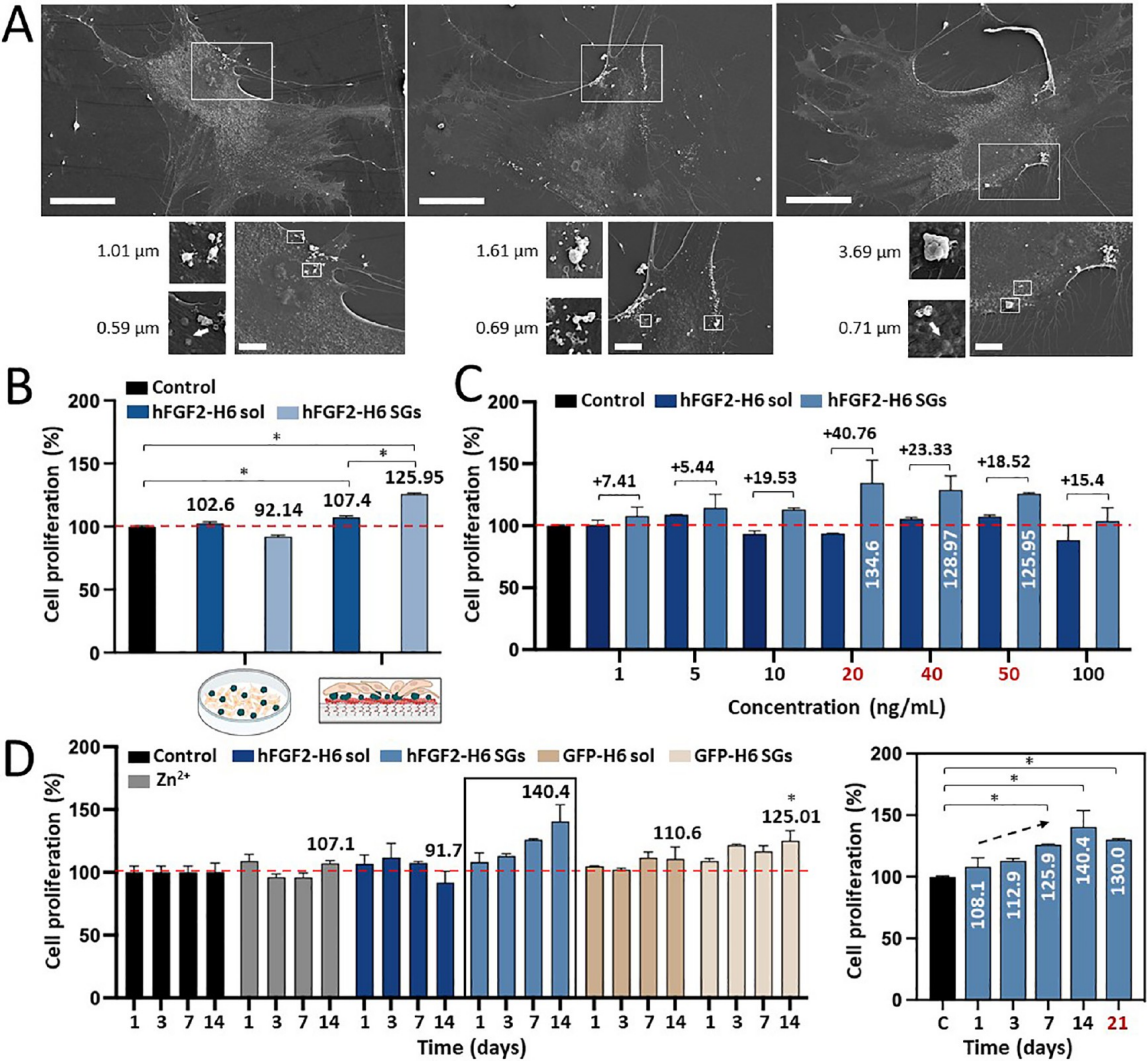
MSC proliferation and interaction with hFGF2-H6 SGs PEA–FN surfaces. (A) Imaging of MSCs by SEM in the presence of hFGF2-H6 SGs (white dots). The white bars refer to 50 μm, and white squares display the framed regions in the insets (down). The white bars in the insets refer to 10 μm. At the bottom left, close-up pictures of hFGF2-H6 SGs with size numbers. White arrows indicate the measured SGs. (B) MSCs proliferation analysis (in percentage) upon exposure to hFGF2-H6 soluble (dark blue) and artificial SGs (pale blue) samples at 50 ng/mL for 7 days. The left legend corresponds to the seeding gold standard technique and the right legend to the PEA–FN surfaces. The dashed red line displays the 100% proliferation threshold. Peak numbers correspond to each cell’s proliferation percentage. (C) MSCs proliferation analysis (in percentage) upon exposure to soluble hFGF2-H6 (dark blue) and artificial hFGF2-H6 SGs (pale blue) at increasing concentrations (from 1 to 100 ng/mL) for 7 days. The dashed red line displays the 100% proliferation threshold. Peak numbers correspond to the increased percentage of cell growth comparing hFGF2-H6 SGs with soluble hFGF2-H6. The concentrations of hFGF2-H6 SGs rendering the highest cell growth (namely, 20, 40, and 50 ng/mL) are displayed in red, and the specific growth percentages are indicated as white numbers. (D) MSCs proliferation analysis (in percentage) upon exposure to soluble hFGF2-H6 (dark blue), hFGF2-H6 SGs (pale blue), free Zn^2+^ (gray), soluble GFP-H6 (dark brown), and GFP-H6 SGs (pale brown) at 50 ng/mL for 1, 3, 7, and 14 days. The pointed red line displays the 100% proliferation threshold. Peak numbers correspond to the cell proliferation percentage on day 14. On the right, the same squared cell proliferation graph with an additional time point (day 21) is highlighted in red. Control of MSCs is displayed in black in all cases. Data are expressed as mean ± standard error of the mean (SEM), and the statistical significance achieved when *p* < 0.05 is represented as (*) compared to the cell control.

These hFGF2-H6-based SGs were subsequently moved toward the functionalization of PEA–FN surfaces (Figure 2G top), with the hypothesis that released hFGF2-H6 would then be exposed to the MSCs in a solid phase (Figure 2H). Preliminary adsorption studies revealed micrometric particles as enhanced interactors with FN surfaces (∼20% increase) compared to their soluble counterparts, a fact that seems to be independent of the adsorbed protein and material format (Figure 2G bottom). This fact was indicative of the insoluble nature and tendency to precipitate the SGs. Human MSCs, when cultured on these functional surfaces, showed good adhesion and interaction with the attached granules (Figure 3A). Cell proliferation increased (determined at 7 days) when the GF was used in the particulate format when compared to the standard soluble format used with the PEA–FN surfaces (Figure 3B). This difference was observed within a wide range of tested protein concentrations, among which the concentrations between 20 and 50 ng/mL promoted the highest cell activity on the hFGF2-H6 SG PEA–FN systems (Figure 3C). The stimulation of cell proliferation (∼40% increase when compared to the cell control) was evidenced for at least up to 14 days at 50 ng/mL (Figure 3D left). It is notable that the topography itself (provided by biologically irrelevant, control GFP SGs) had a small but significant positive effect on proliferation, which is lower than that induced by FGF release (Figure 3D). Proliferation was seen to decline after a longer culture time, likely since the cells become confluent on the hFGF2-H6 SG PEA–FN system (Figure 3D right).

**Figure 4:**
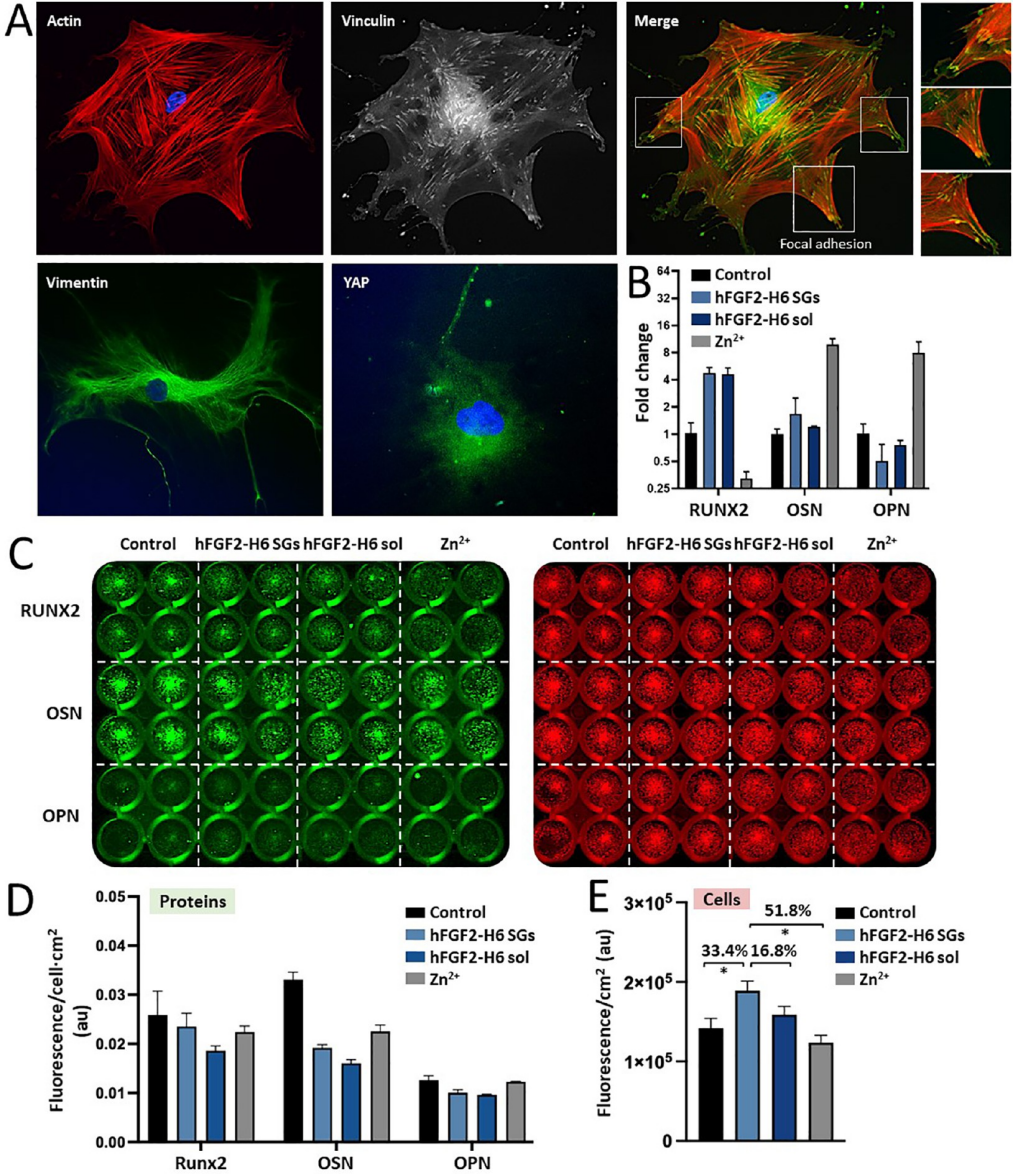
MSCs differentiation in cultures over artificial hFGF2-H6 SGs PEA–FN surfaces. (A) Imaging of MSCs by fluorescence microscopy in the presence of artificial hFGF2-H6 SGs at 50 ng/mL for 14 days. Actin, vinculin, vimentin, and YAP were selected as cell markers. Merge refers to actin and vinculin combined fluorescence signals. White squares highlight cell focal adhesions. Close-up pictures of focal adhesions are displayed on the right panels. (B) Fold change on mRNA content (meaning RUNX2, OSN; osteonectin, and OPN; osteopontin gene expression) in MSCs upon incubation with soluble hFGF2-H6 (dark blue), artificial hFGF2-H6 SGs (pale blue), and free Zn^2+^ (gray) at 50 ng/mL for 14 days. (C) In-cell Western (ICW) immunodetection of RUNX2, OSN, and OSP proteins in MSCs extracts upon incubation over soluble hFGF2-H6, artificial hFGF2-H6 SGs, and free Zn^2+^ (gray) at 50 ng/mL for 14 days. The protein signal is displayed in green, and the cell signal is in red. (D) Statistical analysis of protein signal (green from panel C) expressed as fluorescence per cell and cm^2^ in absorbance units). (E) Statistical analysis of cell signal (red from panel C) expressed as fluorescence per cm^2^ in absorbance units (au). Peak numbers correspond to the increased percentage of cell growth comparing artificial hFGF2-H6 SGs (pale blue) with soluble hFGF2-H6 (dark blue), free Zn^2+^ (gray), and control MSCs (black). Data are expressed as mean ± SEM, and statistical significance is achieved when *p* < 0.05 is represented as (*). Control refers to MSCs seeded on top of FN-PEA surfaces.

The MSC phenotype was then analyzed on the hFGF2-H6 SG-based surfaces (50 ng/mL and 14 days) via immunofluorescence. Actin and vinculin signals indicated good adhesion and spread cell morphology, confirming a well-established cytoskeletal structure (Figure 4A). Vimentin, an intermediate filament protein associated with MSCs, was seen to be well organized in MSCs on hFGF2-H6 SG-based surfaces. Finally, the moderate perinuclear expression of Yes-associated protein (Yap) suggested mechanotransduction-induced differentiation, which was not seen in MSCs on the hFGF2-H6 SG-based surfaces (Figure 4A).

Several osteogenic-related genes were then investigated to determine whether differentiation of the MSCs could be observed. Also, reporter mRNA (mRNA) expression was analyzed or genes encoding the runt-related transcription factor 2 (RUNX2), osteonectin (OSN), and osteopontin (OPN). RUNX2 is an early-stage transcription factor mainly involved in osteoblast differentiation, while osteonectin and osteopontin are late-stage differentiation markers, expressed as bone mineralization occurs.^38^ A trend of FGF (soluble and in SG format) increasing RUNX2 was seen (Figure 4B). However, this was a very late expression of RUNX2, and so, it could show a lag expression not linked to differentiation.^39^ For the later osteogenic markers, OSN and OSP, no change to the control was observed (Figure 4B). The free zinc control appeared to be the most potent differentiation initiator (Figure 4B).

Similar trends were observed upon detecting the in-cell expression of respective proteins (RUNX2, OSN, and ONP) with very little evidence of differentiation (Figure 4C). When analyzing the proliferative profile of each condition by measuring the expression of cell tag (proportional to cell number), very similar outcomes were detected as in Figure 4D, indicating that the higher proliferative inductors (especially hFGF2-H6 SGs with up to a 33.4% increase) promoted a slightly lower level of differentiation (Figure 4B,D) and vice versa (Figure 4E). This fact suggests that the GF systems (sol and SG) both drive growth without differentiation, with this effect being significantly enhanced in the case of the hFGF2-H6 SG PEA–FN-based surfaces.

**Figure 5:**
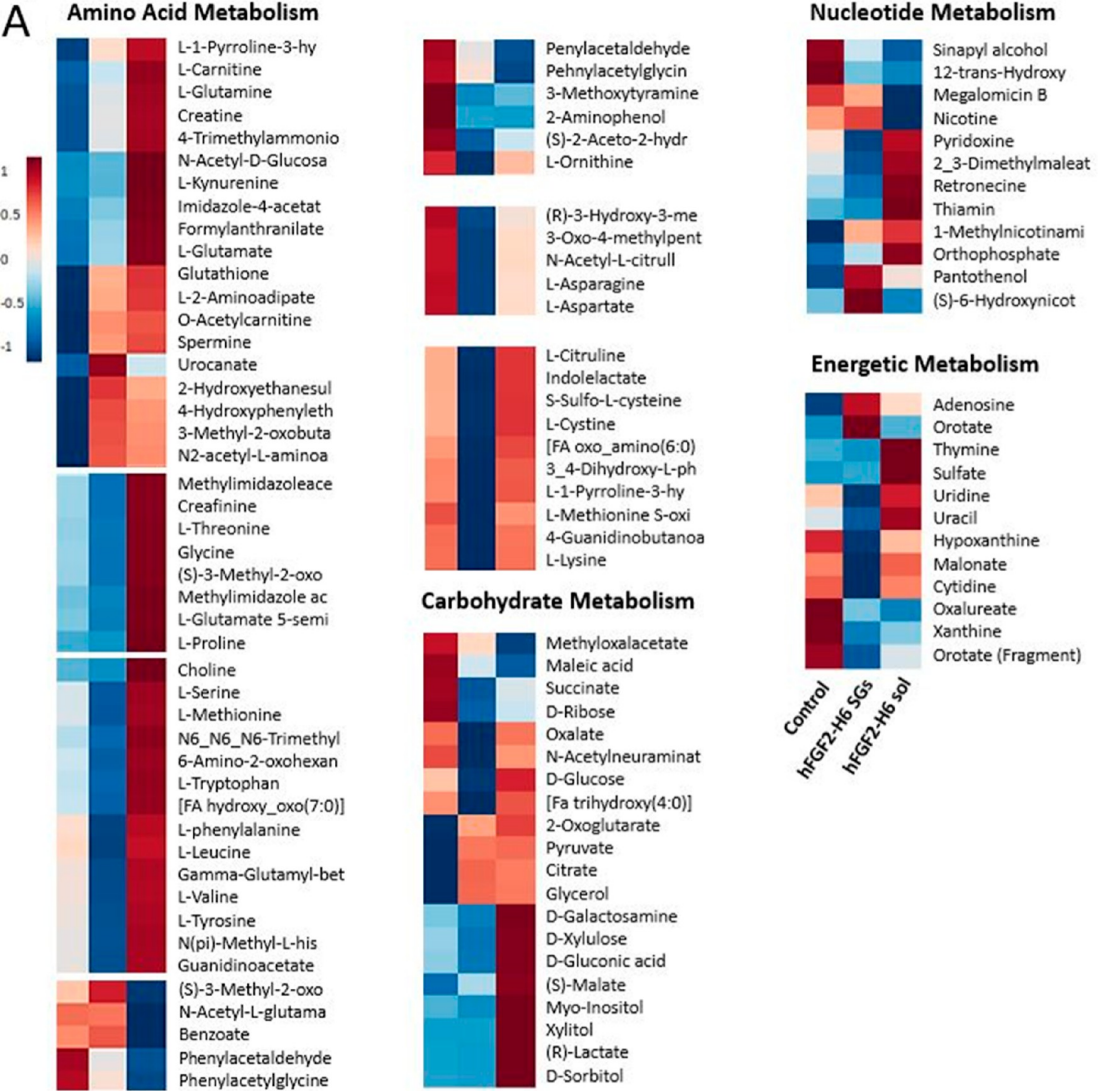

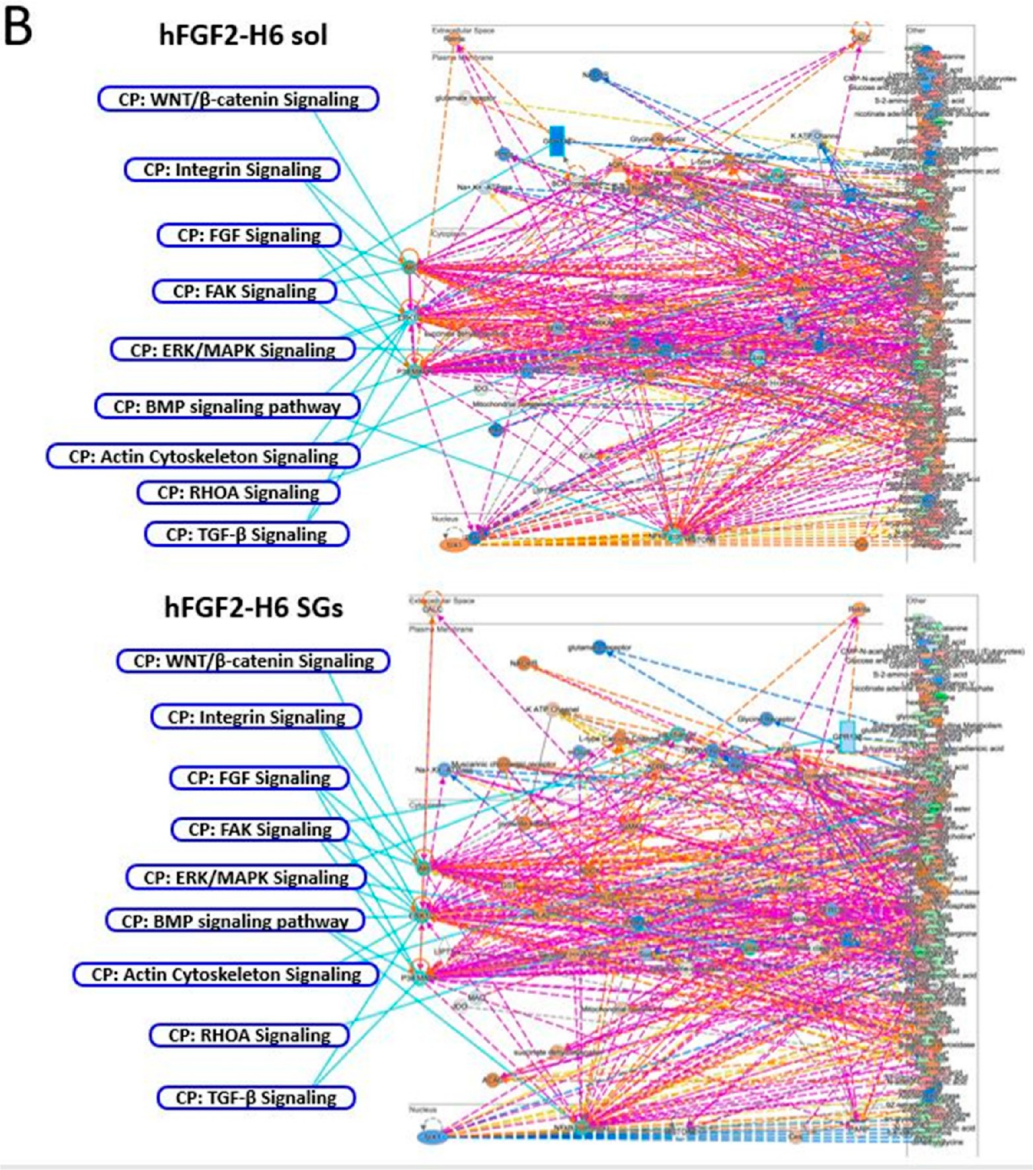
Analysis of metabolomic and signaling pathways in MSCs upon incubation with soluble hFGF2-H6 or SGs. (A) Metabolite heat maps show an abundance for metabolites involved in amino acid, carbohydrate, nucleotide, and energy metabolism. hFGF-H6 SGs tended to produce downregulation in these pathways. (B) Network analysis linked to biochemical activity prediction shows metabolites interacting with major biochemical hubs Akt, ERK 1/2, and p38 MAPK, with predicted up-regulation of ERK 1/2 seen on hFGF2-H6 SG and p38 MAPK seen on soluble hFGF2-H6. It is interesting to note that despite significantly reduced metabolite abundance in cells on the hFGF2-H6 SG samples, biochemically (looking at all hubs), these cells appeared to be more active.

Next, we employed untargeted metabolomic analysis after 14 days of incubation to better understand the MSC response. Comparing soluble hFGF2-H6 PEA–FN and hFGF2-H6 SGs PEA–FN to a standard control culture and considering metabolites involved in the DNA/RNA metabolism (nucleotides), respiration (carbohydrates), protein synthesis (amino acids), and energy, a trend of identification of fewer relevant metabolites was observed, especially within the energetic metabolism analysis (Figure 5A). Noteworthily, all these pathways are considered important in MSC differentiation. Indeed, MSC differentiation is defined by an increase in mitochondrial respiration (oxidative phosphorylation) due to increased energy demand and the increased expression of phenotypical proteins.^40,41^

Taking the metabolite identifications into Ingenuity Pathway Analysis, which allows evaluation of cell signaling networks and pathways, we created large-scale networks from the top 5 identified signaling networks recognized from soluble hFGF2-H6 PEA–FN vs control and hFGF2-H6 SGs PEA–FN vs control. The same precise metabolites and networks were flagged for both hFGF2-H6 conditions, with subtle differences. To explore these differences, we used the molecular prediction tool that associates changes in metabolite patterns with changes in biochemical signaling identified through the literature. Further, we selected a number of biochemical pathways (Figure 5B) known to be involved with MSC growth and differentiation, including those involved in adhesion (integrin signaling, focal adhesion kinase, cytoskeleton (RhoA signaling, actin cytoskeleton), mitogen-activated protein kinases ((MAPK), extracellular signal-related kinase 1/2 (ERK 1/2)) and GFs (FGF, bone morphogenetic protein (BMP), transforming GF, and also wnt/b-catenin signaling.^33,42−44^

These pathways were seen to link into three main signaling hubs: protein kinase B (Akt), ERK 1/2, and p38 MAPK. Akt is known to have roles in cell survival and growth. ERK 1/2 is a mitogenic switch between growth and differentiation that is classically implicated in proliferation but that, under certain conditions, can activate transcription factors such as RUNX2. Finally, p38 MAPK is implicated in MSC differentiation. It was seen that for soluble hFGF2-H6 PEA–FN, ERK 1/2 was predicted to be unchanged from control with p38 MAPK upregulated (Figure 5B). For hFGF2 SGs PEA–FN, p38 MAPK was predicted to be attenuated, and ERK 1/2 was upregulated (Figure 5B). It is interesting that while metabolite abundance in MSCs on SGs PEA–FN with hFGF2-H6 was lower, the biochemical pathways that regulate growth are predicted to be higher. It is probable that the cells need to down-regulate a lot of metabolic routes (e.g., oxidative glycolysis) in order to avoid differentiation and aging. Therefore, for controlled growth, it is likely that more targeted regulations are used to prevent these unwanted phenotypes.^40^ This data is, again, in agreement with the MSCs on hFGF2-H6 SGs PEA–FN, which had enhanced growth without differentiation.

## Discussion

Tissue engineering seeks to create artificial structures that mimic the natural ECM^45^ and that benefit from the functional and structural protein versatility achievable by genetic engineering (i.e., the generation of chimeric proteins).^46^ Among the clinically appealing protein materials found in nature, SGs from the human endocrine system are functional amyloids, at the microscale, that release a large set of peptide hormones for the regulation of the cellular and systemic milieu.^16,47,48^ These functional amyloids act as both protein reservoirs and protein-releasing structures (i.e., for prolactin and growth hormone).^48,49^ In this context, we have explored here the manufacturing and performance as functional topographies of synthetic versions of SGs releasing a recombinant hFGF2-H6,^30^ a well-known GF for MSCs,^50,51^ and how these materials could be properly applied to induce changes in the behavior of human MSCs. Despite their potential in regenerative medicine, artificial SGs have never been exploited as cell microenvironment enhancers of cell responses upon release of GFs and over PEA-organized FN surfaces.

Several studies have examined the feasibility of naturally occurring IBs (biochemically related amyloidal clusters) to achieve topographical targeted osteogenesis.^29,52^ However, these protein aggregates, produced in vivo in recombinant bacterial cells, still exhibit major biocompatibility and homogeneity issues because of their natural origin and heterogeneous and batch-to-batch variable composition.^53^ What is more, the development of hFGF2-H6-based SGs-FN interfaces was also motivated by the structural properties of the ECM and the ability of FN to amplify the adsorbed GF potency.^33^ This innovative approach leverages an H6 tag to confer uncomplicated zinc-mediated clustering properties onto the functional protein, here hFGF2-H6, while having a minimal impact on its structure and function. Simultaneously, this method increases the solubility and thermal stability of the proteinaceous entities due to an anticipated polypeptide rearrangement.^37^ The resultant fusion construct was produced and purified (up to 99.3% purity) and free from contaminating DNA (Figure 1). Upon increasing concentrations of cationic Zn (up to 10 mM), the H6-tagged GF clustered as discrete, insoluble micromaterials of around 1 μm in size, mimicking the human SG system.^21^ These granular entities gradually released full-length protein into the soluble fraction over at least 7 days, organized as homo-oligomers within the nanoscale (11 to 20 nm; Figure 2).

Interestingly, the hybrid platform consisting of hFGF2-H6 SGs and PEA–FN surfaces promoted faster cell proliferation than plain soluble hFGF2-H6 (up to 20%) at defined protein concentrations (from 20 to 50 ng/mL). This fact suggests the topographical adequacy of disintegrating SGs for MSCs growth under sustained release of the active GF, which probably reaches more steady levels than the plain soluble protein, also in agreement with the enhanced proteolytic stability observed in the SG version (Figure 2D). The stimulation of cell proliferation was observed up to 14 days, while at day 21, in line with an expected highly degraded state of SGs, the culture reached a plateau, as the cells became confluent, and this can cause initiation of differentiation (Figure 3).^54^

The subsequent MSC phenotype analysis revealed a well-established cytoskeletal structure, focal adhesion morphology, and growth profile for cells on hFGF2-H6 SGs and PEA–FN surfaces.^55,56^ In addition, while moderate perinuclear expression of Yap (Figure 4A) was seen in cells on hFGF2-H6 SGs and PEA–FN surfaces, intranuclear Yap was not observed, potentially suggesting only limited differentiation.^57^ The analysis of osteogenic-related gene expression provided information on the initial phases of MSC differentiation toward this osteogenic lineage^58^ as well. Importantly, there were no statistically significant differences in gene expression between soluble hFGF2-H6 and SG reservoirs. This finding indicates that the material format does not play a role in promoting cell differentiation. Additionally, it is worth noting that free ionic Zn deposited on this topography was the most osteogenic condition that we tested, and also, it had been previously noted as osteogenic.^59^ Similar findings were observed in the in-cell protein expression analysis, validating the capability of the system to maintain increased amounts of cells without differentiation, particularly for hFGF2-H6 SGs, in comparison to their soluble counterpart (Figure 4).

The use of SGs to release hFGF2-H6 onto the PEA–FN surface that absorbs GFs likely provides an advantage, as the release maintains the hFGF2-H6 pool to provide enhanced effects over a single application of hFGF2-H6. The study is further interesting as previous reports using PEA–FN have used BMP-2 to drive osteogenesis^32,33^ with significantly greater efficiency to soluble BMP-2 administration at higher doses. The current study, therefore, shows that the effect of other GFs, here hFGF2-H6, is not changed by the PEA–FN interface but is potentiated. In addition, MSC growth is an important facet to control. GMP cell manufacturers need to be able to grow millions of MSCs (∼20–100 million) per dose^60,61^ for clinical trials and delivery of products. However, MSCs can senesce and age in culture, and so, it can be a challenge to achieve MSC numbers for larger-scale clinical trials.^62^ Simple coating systems, such as PEA, linked to FGF2 delivered by SGs can help us achieve larger MSC numbers.

Finally, although the present study presents a specific setup for the efficient delivery of hFGF2-H6, it represents, as a whole, an important and generic proof of concept regarding the use, tailoring, and application of the SG format to the effective release of bioactive proteins in regenerative medicine. It must be noted that more than one single bioactive protein could be combined in a multiple display system, which might allow the fast exploration of new synergies (or the full exploitation of the known ones) in the protein-based control of cell growth and differentiation.^63−68^ Even the proposed platform is highly promising, the right selection of the particular GF, its ability to aggregate as His-tagged forms as leaking material, and the insolubility of the SGs leading to background adsorption might represent potential limitations for a straightforward application, which would involve a tailored design for specific purposes. Apart from that, the simplicity of the SG fabrication,^37^ the lack of in vivo toxicity,^69^ the full biological activity retailed by the building block polypeptide, even structurally complex,^28^ and the time-sustained release of the embedded proteins (combined with the mechanical stability of SGs)^70^ make the properties of the SG platform transversal and especially suited as a novel tool to control and regulate mammalian cell growth.

## Conclusions

A novel hybrid tissue engineering-oriented platform has been designed, converging both the human artificial SG and ECM FN-derived systems. The resultant dynamic functional biomaterial releases bioactive hFGF2-H6 nanoparticles in a time-sustained way, and its application in tissue engineering has been validated here for the first time by the culture of MSCs. In this context, the hybrid platform is able to trigger high levels of MSC proliferation while preventing progression into an osteogenic-related differentiation state. This potential can have specific implications in the capability to culture, at large-scale, naive MSCs for, e.g., cell therapy. Growing large numbers of cells that do not senesce and that do not differentiate is critical to the ability of start-up companies to sell into healthcare systems.^62^ The engineering of the involved material is simple, and the in vitro fabrication of SGs from pure protein ensures chemical homogeneity and batch-to-batch consistency. Therefore, we propose the secretory/solid phase presentation approach of this particular GF as a choice option in the enhanced MSC manufacture and the whole system as a transversal, versatile, and generic platform to control and regulate cell proliferation under different settings. Beyond the precise application described here, the universal concept underlying the proof of concept is the production of synthetic SGs. They are emerging microscale protein materials suited for the release of protein drugs (GFs, hormones, and others, in a functional form) resulting from simple fabrication processes. Based on metal-protein coordination, they show full applicability in regenerative medicine and the regulation of cell growth and differentiation.

## Materials and Methods

### Genetic Design, Production, and Purification

The genetic sequences coding the histidine-tagged humanized FGF2 version (low molecular weight, approximately 18 kDa, and UniProt code: D9ZGF5_HUMAN; hFGF2-H6) along with control GFP-H6 were designed in-house, tacked on a pET22b plasmid using *Hin*dIII and NdeI restriction enzymes, and obtained from GeneArt (Thermo Fisher Scientific). Recombinant protein production and purification were achieved using *E. coli* as a cell factory, as delineated elsewhere.^30^

### Purity, Integrity, Concentration, and DNA Content

The physicochemical analysis of the obtained proteins was performed following the previously reported protocols.^71^ The procedures included SDS-PAGE and Bradford staining to estimate protein purity, disintegration, structure, and concentration. Protein integrity was assessed via MALDI-TOF, and primary sequence properties were diagnosed using the ProtParam web tool (hosted by ExPASy). The bicatenary DNA (bcDNA) content was measured by a Nanodrop One System (Thermo Fisher Scientific) and expressed as the absorbance ratio *A*_260_/*A*_280_.

### Size and Surface Charge Determination

The protein VSD (in nanometers) and ζ-potential (in mV) were assessed by dynamic light scattering (DLS) and electrophoretic light scattering, respectively. The measurements were performed at a standard temperature of 25 °C or increasing temperatures (from 10 to 50 °C) and a wavelength of 633 nm using a Zetasizer Advance Pro instrument (Malvern Instruments). Fast mode (meaning run: 0.839 s) was solely utilized during the measurements of SGs and intensity values collected. VSD was selected to represent the hydrodynamic protein size, as it more accurately reflected, in this particular hFGF2-H6 protein case, the predominant nanoparticle population compared to intensity data (also shown in captions Figure 1F for comparison), which can be biased by a minor population of large particles (intensity values C: 133.1 ± 2.01 nm, zinc: 155.58 ± 10.70 nm, and EDTA: 175.27 ± 10.22 nm), representing less than 1% of the total protein population, that scatter the majority of light. Ten replicates were used to calculate the final averaged size, including error values. Respective PDI values are additionally displayed.

### Morphometric Characterization of Nano- and Microstructures

Electron microscopy images of nanosized protein materials were taken by TEM following previously outlined procedures for both imaging and sample preparation.^72^ Microsized structures were imaged using the FEI Magellan 400L XHR SEM operating at 20.00 kV, with magnifications set at 8000 and 20,000. During imaging, simultaneous STEM (TEM images) and TLD (SEM images) detectors were utilized.

### Fabrication of SGs

The purified and soluble protein was initially adjusted to 2 mg/mL into fixed final volumes of 250 μL. Then, a filtered solution of ZnCl_2_ (at 400 mM) was added in a precise 10 mM final concentration. The resulting solutions were gently mixed, incubated for 10 min at room temperature, and then subjected to centrifugation at 10,000*xg* for 5 min to isolate the insoluble and soluble fractions. The remaining soluble protein was quantified by a Bradford assay for an accurate calculation of the precipitated protein. Finally, the protein pellets (namely, SGs) were stored at −80 °C for postponed use.

### Protein Release from SGs

The release of soluble protein was monitored at 37 °C for 1, 3, and 7 day time points following a previously reported procedure.^24^

### Fabrication of FN-PEA Coverslips/24-Well Plates and Protein Adsorption

Well plates, glass coverslips, and plastic Thermanox were coated with PEA using a custom-build capacitively coupled plasma reactor, following the protocol described elsewhere.^33,73^ Substrates were then coated with FN (20 μg/mL) for 1 h in both systems before GF coating. Quantification of hFGF2 and GFP-H6 nanoadsorption over FN-PEA surfaces was measured via a His-Tag ELISA Kit (Cayman Chemical) following the manufacturer’s instructions and indirectly calculated upon soluble fraction protein analysis. 24-well plates were used for long-term experiments (meaning 14 days) and coverslips for short-term. Note that 24-well plates were used as the default analysis system as they reduced experimental complexity, increased robustness, and improved workflow, whereas glass coverslips were only used to ease sample handling during microscopy imaging. Plastic Thermanox was only used for SEM analysis to avoid the PEA coating peeling off after osmium staining and the progressive ethanol dehydration involved in SEM analysis.

### Morphometric Characterization of FN-PEA/FN Surfaces

AFM was used to determine whether the FN nanonetwork spontaneously assembled on PEA surfaces. A 200 μL droplet of FN was placed on the surface of glass coverslips treated with PEA and allowed to adsorb for 10 min. Afterward, the liquid was removed from the surface and washed twice with DPBS, followed by a final wash with Milli-Q water. The coverslips were then dried under a stream of nitrogen before AFM imaging. Imaging was performed using a JPK Nanowizard 4 (JPK Instruments) apparatus in tapping mode, acquiring both height and phase images. Image analysis was conducted using JPK Data Processing software, version 5.

### Cell Culture and MSCs Seeding

Human bone marrow MSCs (PromoCell) were cultured in α-minimum essential medium containing 10% fetal bovine serum (FBS), 1% penicillin/streptomycin, 1% fungizone, 2 mM l-glutamine, and FGF-2 (1 ng/mL) at 37 °C in a 5% CO_2_ atmosphere. Cells were maintained at a density of 10^4^/cm^2^ in T-75 flasks using high-glucose Dulbecco’s modified Eagle’s medium with 1% penicillin/streptomycin and 2% FBS and changed twice a week. Passages P_0_ to P_5_ were used for all of the experiments.

### Proliferation Assays

Prior to protein incubation, 2x10^3^ atmosphere. Cells were maintained at a density of 104/cm2 cells/cm^2^ were seeded onto protein-FN-PEA-functionalized 24 well plates for 2 h using 2% FBS-supplemented Eagle’s medium to support cell adhesion, being increased up to 10% FBS for cell growth. Cell proliferation was monitored via AlamarBlue (Bio-Rad) assay, and absorbances 570–600 nm were measured in a Dynatech MR700 plate reader following the manufacturer’s instructions.

### Morphometric Characterization of MSCs over SGs-FN-PEA Surfaces

MSCs seeded (2x10^3^/ cm^2^) on top of SGs-FN-PEA plastic coverslip surfaces were initially fixed with 1.5% glutaraldehyde for 1 h for cellular and protein structure preservation. Then, cells were repetitively washed with rinse buffer and stained for 1 h with 1% osmium tetroxide and, later on, 0.5% uranyl acetate as contrast enhancer agents. Progressive ethanol dehydration was enacted prior to gold coating (of around 10–20 nm thickness) using a Quorum High Vacuum Q150T coating system. Samples were viewed on a JEOL IT100 SEM running at 10 kV, and TIF images were captured using Intouch Scope version 1.05 software (Figure 3A). Note that plastic coverslips were used to improve cell surface adherence upon osmium staining and progressive ethanol dehydration. If glass coverslips were used, cells were peeled off, and subsequent imaging was compromised.

### Immunofluorescence Staining for MSCs Differentiation

Cells were cultured for 14 days and fixed on protein-FN-PEA-functionalized glass coverslips following procedures described elsewhere.^33^ Primary monoclonal antibodies (Ab) against vinculin (1:400; Sigma-Aldrich), vimentin (1:200; Sigma-Aldrich), and Yap (1:200; Sigma-Aldrich) were incubated with rhodamine phalloidin that stains actin (1:300; Invitrogen) overnight at 4 °C in 1% BSA/DPBS. After several washing steps (5 min each) in PBS/0.5% Tween, a secondary antimouse biotinylated Ab (1:50; Vector Laboratories) was incubated for 1 h at 37 °C. Samples were washed as previously noted, and streptavidin-fluorescein (FITC) Ab (1:50; Vector Laboratories) was incubated for 30 min at 4 °C for signaling purposes. Samples were finally rinsed in PBS/0.5% Tween and mounted with Vectashield containing DAPI staining (Vector Laboratories). A Zeiss fluorescence microscope was used for imaging, and pictures were captured at 20× magnifications (Figure 4A).

### Detection of Expressed Osteogenic-Related Genes

Cells were cultured for 14 days on protein-FN-PEA-functionalized 24-well plates, and RNA was extracted as described elsewhere using a Qiagen RNeasy micro kit (deoxyribonuclease treatment included) following manufacturer’s instructions, and RNA’s quantity and integrity were measured via NanoDrop (Thermo Fisher Scientific). Quantitative PCR (qPCR) was used for Runx2, osteonectin, and osteopontin expression analysis, and GAPDH was utilized as a housekeeper gene (a standard for normalization). RNA samples were reverse transcribed using a QuantiTect Reverse Transcription Kit (Applied Biosystems) and qPCR executed by the SYBR Select Master Mix (Life Technologies) following manufacturer’s instructions and detected via the 7500 Real-Time PCR System (Applied Biosystems; Figure 4B). The comparative cycle threshold method was used for gene expression quantification and displayed as fold change (*n* = 3 per group).

### Detection of Expressed Osteogenic-Related Proteins (ICW Blot)

Cells were cultured for 14 days and fixed on protein-FN-PEA-functionalized 24 well plates, permeabilized, and blocked following previously reported procedures.^33^ Cells were incubated with monoclonal primary Abs (1:200) in blocking buffer (PBS/1% milk protein) at room temperature for 2.5 h, respectively; Runx2 (Santa Cruz Biotechnology, C1319), osteonectin (Santa Cruz Biotechnology, SC398419), and osteopontin (Santa Cruz Biotechnology, B1218). Cells were then repeatedly washed for 10 min (PBS/0.1% Tween 20) and incubated with an infrared-labeled secondary Ab IRDye 800CW (1:800; LI-COR) + CellTag 700 Stain (1:500; LI-COR) + PBS/0.2% Tween 20 mixture at room temperature for 1 h while being shielded from light. Finally, samples were rinsed again and dried on white paper for infrared signal reading using an Odyssey infrared imaging system.

### 3D Protein Structure Modeling and Statistical Analysis

The ColabFold platform,^74^ within the AlphaFold2 algorithm,^75^ was employed to computationally predict the three-dimensional (3D) conformations of folded protein states. The default settings were utilized, and each primary FASTA sequence was used as a query individually for prediction. ChimeraX-1.3 software was used for 3D structure processing and interamino acidic distance calculation (Figure 1A). Statistical analysis was outlined as previously reported^72^ using the GraphPad Prism software, and data was expressed as mean ± SE.

### Metabolomics

Metabolomic data was collected following the procedures described elsewhere.^41^

## Abbreviations

ECM: extracellular matrix
ECM-FN: extracellular matrix fibronectin-derived
GF: growth factor
FN: fibronectin
hFGF: human fibroblast growth factor
H6: hexahistidine
MSC: mesenchymal stromal cell
SG: secretory granules
TEM: transmission electron microscopy
